# Machine learning models for predicting early hemorrhage progression in traumatic brain injury

**DOI:** 10.1038/s41598-024-61739-3

**Published:** 2024-05-22

**Authors:** Heui Seung Lee, Ji Hee Kim, Jiye Son, Hyeryun Park, Jinwook Choi

**Affiliations:** 1 Department of Neurosurgery, College of Medicine, Hallym Sacred Heart Hospital, Hallym University, Anyang-si, Korea; 2 Interdisciplinary Program for Bioinformatics, Graduate School, Seoul National University, Seoul, Korea; 3 Interdisciplinary Program for Bioengineering, Graduate School, Seoul National University, Seoul, Korea; 4 Integrated Major in Innovative Medical Science, Graduate School, Seoul National University, Seoul, Korea; 5 Department of Biomedical Engineering, College of Medicine, Seoul National University, 103 Daehak-ro, Jongno-gu, Seoul, 03080 Korea; 6 Institute of Medical and Biological Engineering, Medical Research Center, Seoul National University, Seoul, Korea

**Keywords:** Traumatic brain injury, Intracerebral hemorrhage, Machine learning, Extreme gradient boosting, Random forest algorithm, Computed tomography, Outcomes research, Brain injuries

## Abstract

This study explores the progression of intracerebral hemorrhage (ICH) in patients with mild to moderate traumatic brain injury (TBI). It aims to predict the risk of ICH progression using initial CT scans and identify clinical factors associated with this progression. A retrospective analysis of TBI patients between January 2010 and December 2021 was performed, focusing on initial CT evaluations and demographic, comorbid, and medical history data. ICH was categorized into intraparenchymal hemorrhage (IPH), petechial hemorrhage (PH), and subarachnoid hemorrhage (SAH). Within our study cohort, we identified a 22.2% progression rate of ICH among 650 TBI patients. The Random Forest algorithm identified variables such as petechial hemorrhage (PH) and countercoup injury as significant predictors of ICH progression. The XGBoost algorithm, incorporating key variables identified through SHAP values, demonstrated robust performance, achieving an AUC of 0.9. Additionally, an individual risk assessment diagram, utilizing significant SHAP values, visually represented the impact of each variable on the risk of ICH progression, providing personalized risk profiles. This approach, highlighted by an AUC of 0.913, underscores the model’s precision in predicting ICH progression, marking a significant step towards enhancing TBI patient management through early identification of ICH progression risks.

## Introduction

Despite reports of poor prognosis and high mortality in patients with severe traumatic brain injury (TBI), there is a lack of consensus regarding hospitalization and follow-up policies for patients with mild to moderate TBI^1,2^. Additionally, the radiological progression of intracerebral hemorrhage (ICH) in TBI patients has been observed in 38–51% of cases, irrespective of the level of consciousness at initial presentation^3^. This diversity presents significant challenges in clinical decision-making. Particularly, it is difficult to determine which TBI patients, who are not comatose upon ER admission and do not require immediate neurosurgical surgery, may need more aggressive monitoring or should be admitted to the intensive care unit for close, short-term observation. Conversely, it is also crucial to identify those who can be safely managed through outpatient follow-up. The absence of comprehensive clinical guidelines complicates the ability to make these critical care decisions, underscoring the urgent need for clear and actionable clinical indicators.

In the field of emergency medicine, acquiring an accurate patient history from individuals with head trauma is frequently challenging due to their compromised mental status. As a result, details regarding the mechanism and severity of the head injury are often insufficient for effectively assessing patient outcomes. This study involved the evaluation of clinical factors identified during the initial assessment, which are linked to the progression of intracranial hemorrhage (ICH) in patients with traumatic brain injury (TBI). In this study, we assessed clinical factors during the initial evaluation that are associated with hemorrhagic expansion in patients with traumatic brain injury (TBI).

With recent advancements in machine learning models that have demonstrated superior performance over classical statistical methods in classification and risk factor analysis of large datasets, we employed both the random forest model and extreme gradient boosting (XGBoost) as machine learning tools^4^. Utilizing the XGBoost model, we developed a predictive model for intracerebral hemorrhage (ICH) progression in patients with traumatic brain injury (TBI). Additionally, based on this model, we identified the most significant factors for ICH progression and propose an in-hospital follow-up protocol following the initial evaluation of TBI.

The aim of this study was to stratify and predict the risk of ICH progression based solely on factors that are clearly visible on CT images performed in the emergency room. Specifically, we hypothesized that certain types of ICH carry a higher risk of progression^3^, and aimed to demonstrate that the contribution to ICH progression risk varies by type of ICH.

## Methods

### Patient data collection and preoperative evaluation

We conducted a retrospective analysis of all patients diagnosed with traumatic brain injury (TBI) at the emergency department of our institute from January 2010 to December 2021. This study received approval from our institutional review board (IRB No. 2023-01-002). All patients underwent initial evaluation using computed tomography (CT). We reviewed patient medical records to collect demographic information, comorbid conditions, and past medical history. Data on the presence of intracerebral hemorrhage (ICH), and the level of consciousness at admission scored by the Glasgow Coma Scale were extracted from medical records.

Radiological data, such as the location and morphological type of ICH, and any changes in its extent, were obtained from the initial head CT scans at admission. The Picture Archiving and Communications System (PACS) (Infinite 3.0) was utilized for analyzing these initial CT scans.

Patients classified as having severe TBI, with a Glasgow Coma Scale score of 8 or less, or those in need of early surgical intervention for conditions like acute subdural or epidural hematoma with significant mass effects, were excluded from the study.

### Classification of ICH types

**Figure 1:**
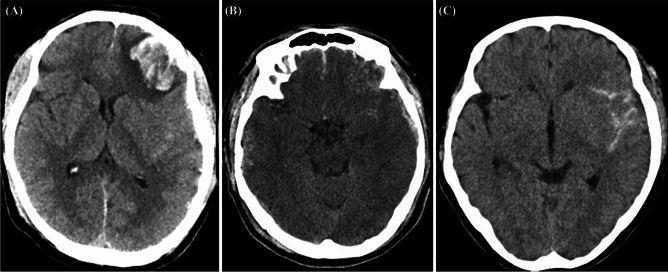
Intracerebral hemorrhage types on CT. (**A**) Intraparenchymal hemorrhage, indicated by uniformly dense lesions within brain tissue. (**B**) Petechial hemorrhage, with characteristic ‘salt and pepper’ patterning alongside subarachnoid hemorrhage. (**C**) Subarachnoid hemorrhage, shown by dense lesions in subarachnoid and sulcal spaces.

ICH types were categorized into three distinct classifications. The first category, intraparenchymal hemorrhage (IPH), was identified by the presence of homogeneously high-density lesions within the brain parenchyma (Fig. 1A). The second category, petechial hemorrhage (PH), was characterized by a ‘salt and pepper’ appearance along with the subarachnoid hemorrhage (Fig. 1B)^3^. The third category, subarachnoid hemorrhage (SAH), was defined by high-density lesions located within the subarachnoid spaces, including the sulcal spaces on the cerebral convexities (Fig. 1C)^5,6^.

### Definition of counter coup injuries

We defined the site of impact in head injury cases by identifying areas of scalp swelling visible on head CT scans. Additionally, other indicators such as skin stapler clips used for scalp lacerations were utilized to pinpoint the impact location. Scalp swelling was characterized by the accumulation of high density in both the subcutaneous and sub-galeal layers^7^.

### Counter coup swelling and fracture

Counter coup swelling was defined as follow:

1. The center of the brain on CT scans was determined as the center of the third ventricle.
2. We measured the angle formed between the center of the scalp swelling and the location of the intracerebral hemorrhage (ICH), relative to the brain’s center.
3. A counter coup injury was identified when this measured angle exceeded 90°^8–10^.

**Figure 2:**
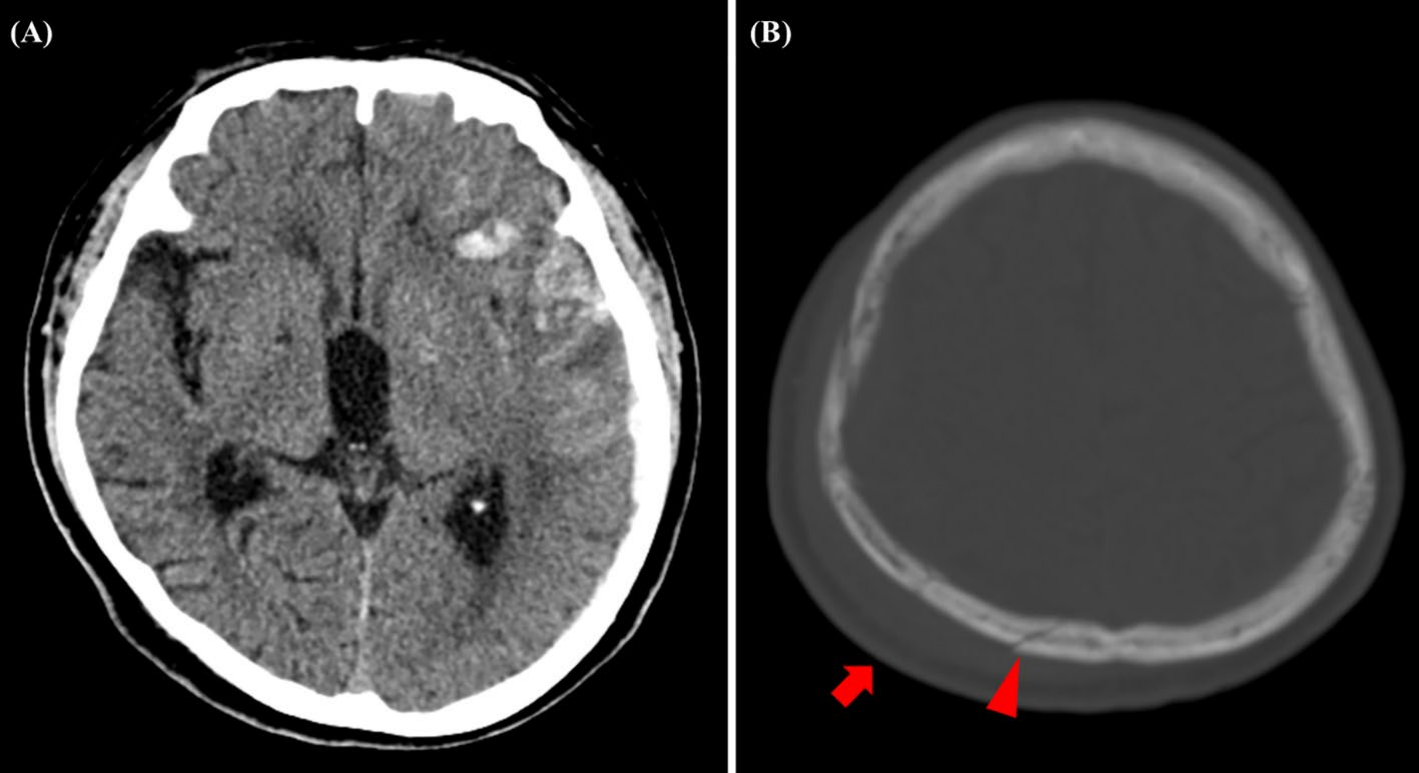
Counter Coup Intracerebral Hemorrhage (ICH) following Traumatic Brain Injury (TBI). (**A**) This CT scan depicts a case of counter coup ICH, where the hemorrhage is observed in the frontal lobe, opposite the site of initial impact, which in this case is the occipital region. (**B**) The corresponding bone window CT image shows a fracture in the occipital bone (indicated by red arrows), which is considered the site of the initial impact.

In cases of counter coup injury, counter coup swelling was specifically defined as the presence of scalp swelling as the sole scalp manifestation. Counter coup fracture was determined when a skull fracture was located beneath the counter coup swelling, as identified on the head CT scan (Fig. 2A and B).

### ICH progression

The primary outcome of our study was the in-hospital progression of intracerebral hemorrhage (ICH). All patients underwent follow-up head CT scans within 12 h of their initial scans. Significant radiological progression of ICH was defined as an increase of 20% or more in the initial ICH area on the first follow-up head CT scans compared to the initial CT scans^11^.

### Statistical analysis

This study was conducted using Python 3.10.5. We assessed data normality using the Kolmogorov–Smirnov test. To identify variables associated with ICH progression, chi-square analysis was performed on categorical variables. Further, multivariate logistic regression analysis was employed to adjust for confounders and to quantify the impact of each significant variable on the risk of ICH progression. Differences between continuous variables were evaluated using the independent t-test. A P-value of less than 0.05 was considered statistically significant.

### Machine learning algorithm and feature importance

We utilized the Extreme Gradient Boosting (XGBoost) algorithm, known for its effective non-linear prediction capabilities and faster computational speed compared to other machine learning methods^12^. The primary outcome for patients was categorized as either ICH progression or non-progression.

The complete dataset from all patients was randomly split into three subsets: training, validation, and test datasets, in a ratio of 8:1:1. These subsets were mutually exclusive. To determine the features for the machine learning model, we extracted the variable importance from the XGBoost model. Additionally, we calculated Shapley Additive exPlanations (SHAP) values to evaluate the impact of each variable on the ICH progression within the test dataset.

All models were developed using Python 3.10.5.

### Evaluation of the performance

To evaluate the performance of our predictive model, we utilized a receiver operating characteristic curve (ROC curve) and calculated the area under the curve (AUC). Additionally, we generated a confusion matrix and computed metrics such as accuracy and the F1-score.

### Ethical approval

All procedures performed in studies involving human participants were in accordance with the ethical standards of the institutional and/or national research committee (name of institute/committee) and with the 1964 Helsinki declaration and its later amendments or.

comparable ethical standards.

### Informed consent

This study was carried out as a retrospective analysis, wherein all patient data were anonymized prior to utilization. The study was initiated after receiving approval from the Institutional Review Board (IRB) of our institution. Furthermore, the requirement to obtain informed consent from the participants was exempted.

## Results

### Data collection and patient characteristics

Data were collected from a total of 650 patients, comprising 465 males and 185 females. The mean age of the cohort was 57.2 ± 19.2 years, with an age range of 18–94 years. Patient demographics and characteristics of the head injury are summarized in Table 1. ICH progression was observed in 144 out of the 650 patients (22.2%). The mean age was significantly lower in patients with ICH progression (56.2 ± 19.5 years) compared to those without (60.7 ± 17.8 years; p = 0.014). Gender distribution did not differ significantly between groups, with males comprising 70.6% of the progression group and 74% of the non-progression group (p = 0.3).

**Table 1:** Patients’ demographics and the characteristics of head injury.

		ICH progression present (N = 144)	ICH progression absent (N = 506)	P-value
Age (years), Mean (SD)	57.2 (19.2)	60.7 ± 17.8	56.2 ± 19.5	0.014
Sex (%)	Male 465 (71.5), Female 185 (28.5)	Male 108 (70.6)	Male 357 (74)	0.3
Glasgow Coma Scale, Mean (SD)	10.9 (1.3)	10.8 (1.3)	10.9 (1.3)	0.2
Current medication				
Antiplatelet	23	4 (2.8)	19 (3.8)	0.4
Anticoagulant	4	2 (1.4)	2 (0.4)	0.24
Type of intracranial hemorrhage				
Intraparenchymal hemorrhage (IPH)	183	39 (27.1)	144 (28.5)	0.75
Petechial hemorrhage (PH)	96	62 (43.1)	34 (6.7)	< 0.001
Subarachnoid hemorrhage (SAH)	351	34 (23.6)	317 (62.6)	< 0.001
Multiple type hemorrhage (≥ 2 ICHs)	30	14 (9.7)	16 (3.2)	0.002
Skull fracture (%)	255 (39.2)	95 (66)	160 (31.6)	< 0.001
Frontal	104	32 (26)	72 (21.4)	0.3
Temporal	51	17 (13.8)	34 (10.1)	0.27
Parietal	25	14 (11.4)	11 (3.3)	< 0.001
Occipital	87	42 (34.1)	45 (13.4)	< 0.001
Impact type of injury (%)				
Counter coup swelling	314 (48.3)	108 (75)	206 (40.7)	< 0.001
Counter coup fracture	139 (21.4)	136 (97.8)	178 (34.8)	< 0.001

### Medication usage

The usage of antiplatelet and anticoagulant medications did not show significant differences between the groups, with antiplatelet use at 2.8% in the progression group versus 3.8% in the non-progression group (p = 0.4), and anticoagulant use at 1.4% versus 0.4%, respectively (p = 0.24).

### Types of intracerebral hemorrhage and skull fractures

Patients with petechial hemorrhage (PH) exhibited a significantly higher rate of ICH progression (43.1%) compared to those without progression (6.7%; p < 0.001). Conversely, subarachnoid hemorrhage (SAH) presented more frequently in the non-progression group (62.6% vs. 23.6%; p < 0.001). The prevalence of skull fractures was also higher in the progression group (66% vs. 31.6%; p < 0.001), particularly occipital (34.1% vs. 13.4%; p < 0.001) and parietal fractures (11.4% vs. 3.3%; p < 0.001).

### Multivariate logistic regression analysis

Our multivariate logistic regression analysis identified several significant predictors of ICH progression, as presented in Table 2. Petechial hemorrhage (PH) significantly increased the risk of progression, with an odds ratio (OR) of 2.52 and a 95% confidence interval (CI) of 1.34–4.76 (p < 0.001). In contrast, subarachnoid hemorrhage (SAH) when present alone appeared to reduce the risk, with an OR of 0.26 and a 95% CI of 0.14–0.46 (p < 0.001). Additionally, the presence of skull fracture significantly increased the likelihood of progression (OR = 4.19, 95% CI: 2.83–6.21, p < 0.001), with countercoup skull fractures (OR = 2.82, 95% CI: 1.51–5.27, p < 0.001) and countercoup swelling (OR = 2.24, 95% CI: 1.2–4.19, p < 0.001) also showing strong associations with increased risk.

**Table 2:** Multivariate logistic regression analysis for independent variables associated with ICH progression.

	Odds ratio	95% confidence interval	P-value
Presence of PH	2.52	1.34–4.76	< 0.001
SAH alone	0.26	0.14–0.46	< 0.001
Multiple lesions (≥ 2 ICHs)	2.2	1.5–3.4	0.002
Skull Fracture	4.19	2.83–6.21	< 0.001
Countercoup skull fracture	2.82	1.51–5.27	< 0.001
Countercoup swelling	2.24	1.2–4.19	< 0.001

ICH, intracerebral hemorrhage; PH, petechial hemorrhage; SAH, subarachnoid hemorrhage.

### Evaluating the importance of effective variables in the predictive model

**Figure 3:**
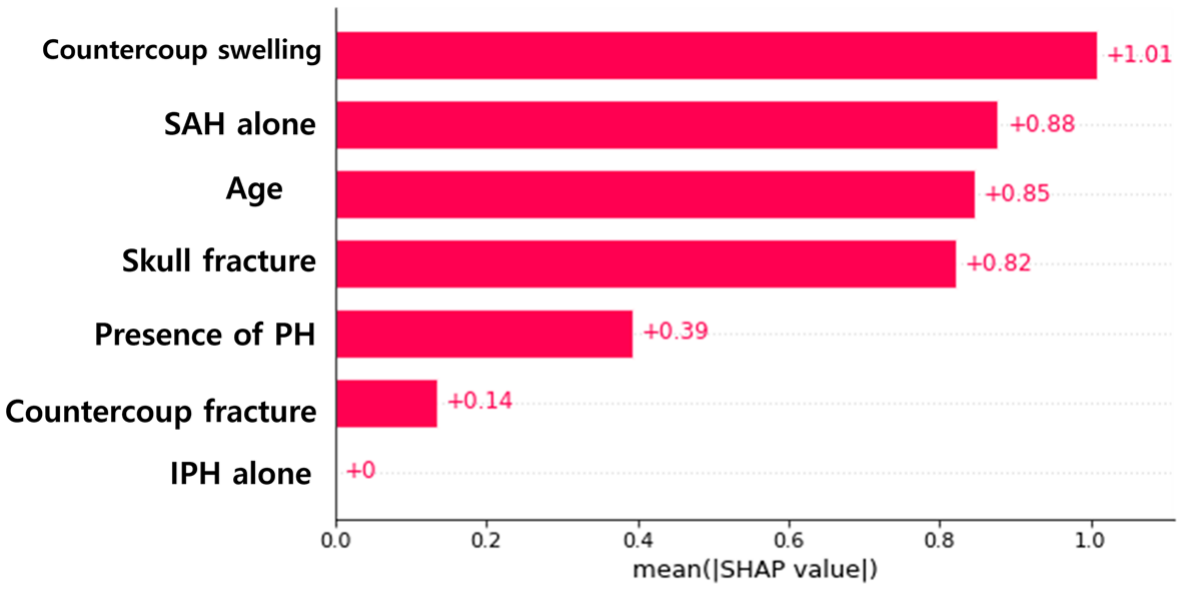
Feature importance for ICH progression prediction. The variables are ranked in descending order of their SHAP (Shapley Additive exPlanations) values, which quantifies the impact of each variable on the model’s predictions. The most influential factors include countercoup swelling, the occurrence of subarachnoid hemorrhage (SAH) alone, patient age, the presence of skull fracture, the presence of petechial hemorrhage (PH), countercoup fracture, and the isolated existence of intraparenchymal hemorrhage (IPH).

The training dataset consisted of 520 patients, while the test dataset included 65 patients. The composition of the training and test datasets is detailed in Table 3. To determine the variables significantly impacting ICH progression, we utilized Shapley Additive exPlanations (SHAP) values. The variables that emerged with the highest SHAP values from the XGBoost model, indicating their significant influence, were, in descending order of SHAP value: countercoup swelling, the occurrence of subarachnoid hemorrhage (SAH) alone, age, presence of skull fracture, presence of petechial hemorrhage (PH), countercoup fracture, and the isolated existence of intraparenchymal hemorrhage (IPH) (Fig. 3).

**Table 3:** Data composition of enrolled TBI patients in the datasets.

	Whole dataset (numbers)	Train set (numbers)	Validation set (numbers)	Test set (numbers)
	Patients	Patients	Patients	Patients
Overall	650	520	65	65
ICH progression				
Present	144	117	12	15
Absent	506	403	53	50

ICH, intracerebral hemorrhage.

### Performance evaluation of the prediction models

**Figure 4:**
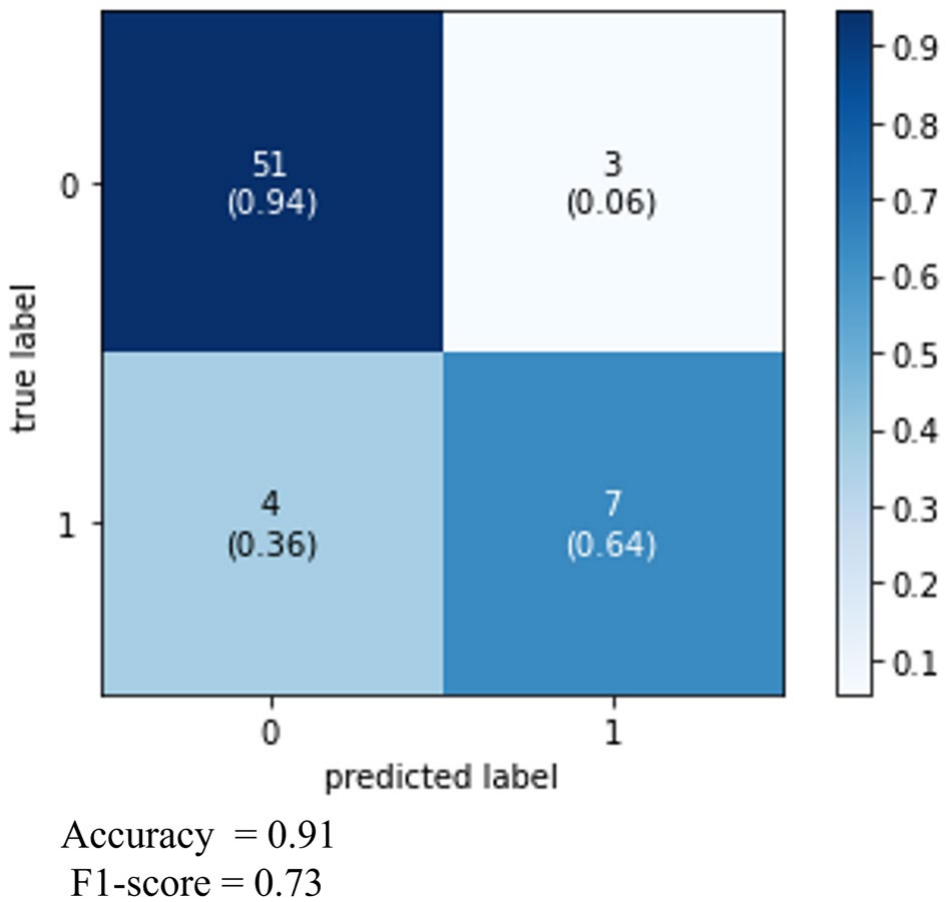
Confusion matrix heatmap for the XGBoost ICH progression prediction model.

**Figure 5:**
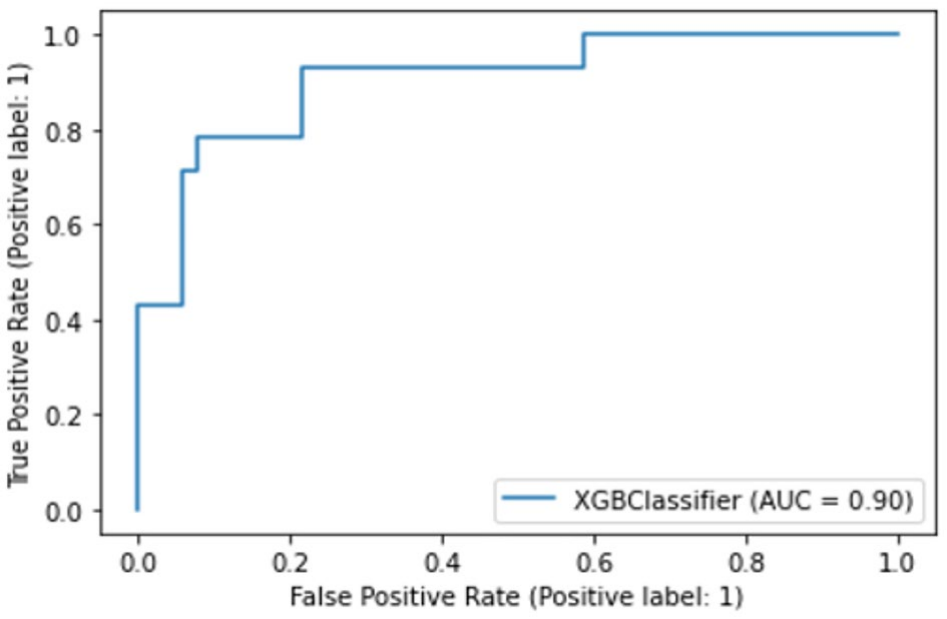
Receiver Operating Characteristic (ROC) curves for binary classification according to Intracerebral Hemorrhage (ICH) progression.

We developed a prediction model for ICH progression using the XGBoost algorithm, incorporating variables with significantly high SHAP values, as derived from the training dataset. Measures of the model's performance, including accuracy, F1 score, and the confusion matrix, are presented in Fig. 4. The model's predictive performance in the test dataset was evaluated based on the area under the curve (AUC) values from the receiver operating characteristic (ROC) curves, with the AUC being 0.9 as presented in Fig. 5.

### Individual risk assessment using effective variables

**Figure 6:**
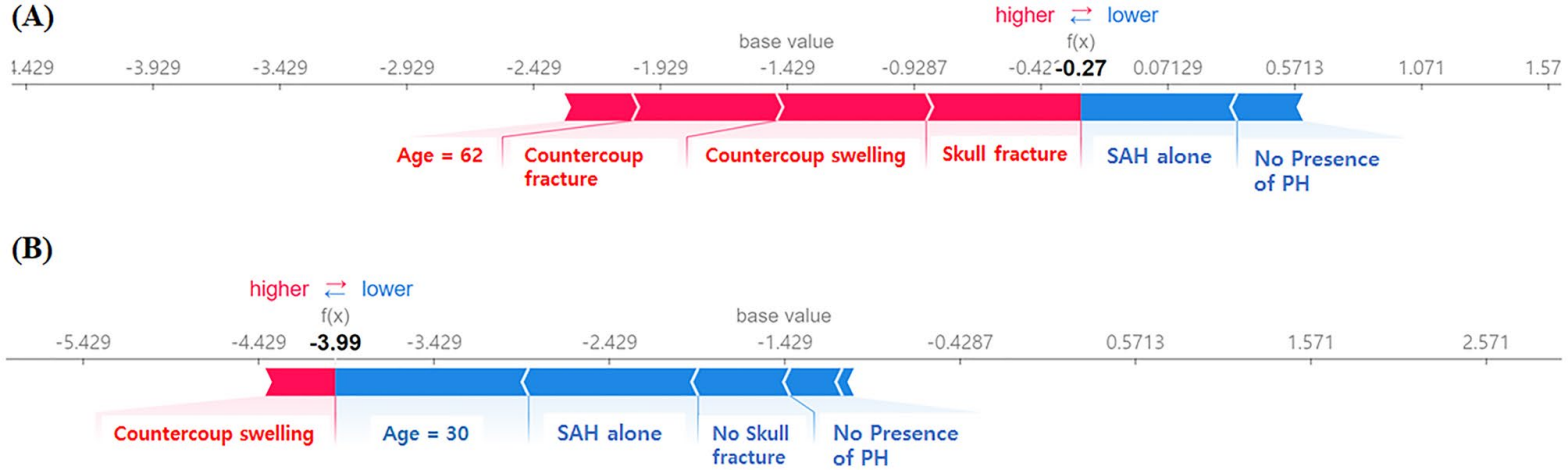
SHAP value-based individual risk assessment diagram that presents visual representation of individual risk assessment for intracerebral hemorrhage (ICH) progression using the XGBoost model and significant SHAP values. The diagram illustrates the influence of each variable on the prediction outcome: red bars extending leftward indicate increased risk, while blue bars extending rightward suggest decreased risk. For example, panel (**B**) shows a longer blue bar for a 30-year-old patient with ‘SAH alone,’ implying a lower risk for ICH progression.

Utilizing the XGBoost algorithm and variables with significant SHAP values, we constructed a diagram for the individual risk assessment of ICH progression. As depicted in Fig. 6, this diagram provides a visual representation of the SHAP value-based risk assessment. Each bar illustrates the impact of a variable on the predicted outcome, with red bars extending leftward to denote increased risk, and blue bars extending rightward to denote decreased risk. For instance, panel B highlights a 30-year-old patient with 'SAH alone,' indicated by a longer blue bar, suggesting a lower risk for ICH progression.

**Figure 7:**
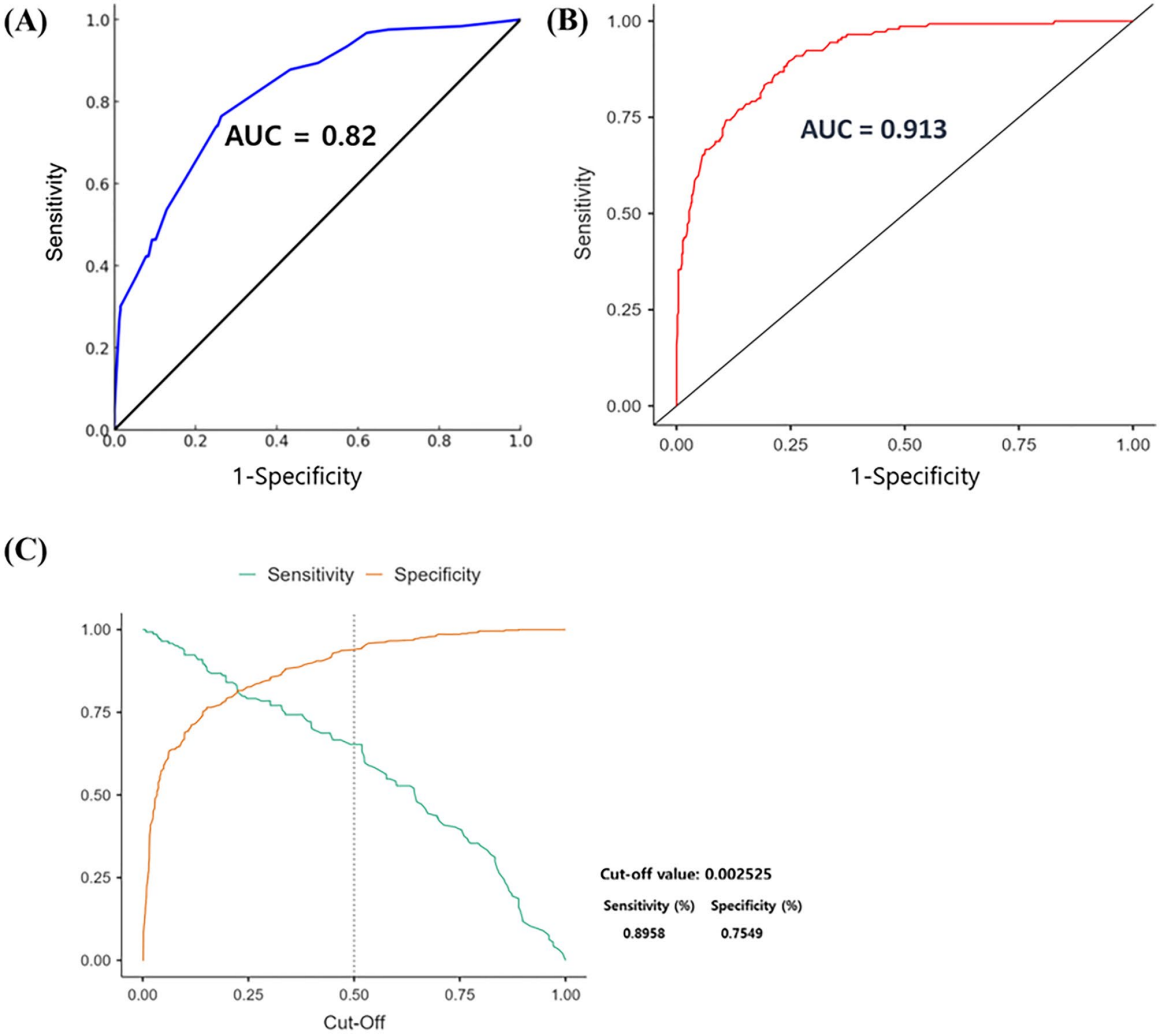
Diagnostic performance of the ICH progression prediction model. (**A**) ROC curve of the multivariate logistic regression model, displaying a blue curve with an AUC of 0.82. (**B**) SHAP Value Cut-Off for ICH Progression Prediction. ROC curve with AUC of 0.913 and sensitivity–specificity curves (**C**), establishing the optimal SHAP value threshold derived from patient data for tailored ICH risk evaluation.

The performance of our predictive models is illustrated in Fig. 7, which assesses model accuracy through different evaluation metrics. Figure 7A displays the ROC curve for the logistic regression model, which achieves an AUC of 0.82, indicating good predictive capability. However, the SHAP value-based model, derived from the XGBoost algorithm, outperforms the logistic regression with an AUC of 0.913 as shown in Fig. 7B. Figure 7C presents sensitivity and specificity curves that identify the optimal cut-off value for predicting ICH progression. This cut-off value, set at 0.002525, provides a sensitivity of 89.58% and a specificity of 75.49%, effectively distinguishing between patients at higher and lower risks for ICH progression.

## Discussion

In emergency situations, the collection of precise clinical information from trauma patients can be challenging, with accurate data often being elusive. Accurately assessing the risk of progression in traumatic intracranial hemorrhage (ICH) is essential, particularly for patients who are relatively stable or exhibit minimal traumatic brain hemorrhage, compared to those immediately identified for emergency surgical intervention among the TBI cohort^13,14^. Additionally, accurately discerning details regarding the mechanism of head injury frequently proves difficult^15^.

In this study, the objective is to create a predictive model for the short-term prognosis of patients with traumatic brain injury. This model emphasizes the use of clear and readily accessible information from the emergency department setting. Specifically, it relies on data from initial head CT scans and findings from physical examinations, both of which are readily available and easily obtained in the emergency room.

### Type of traumatic ICH

Previous literature has explored the analysis of various traumatic ICH types^16^. While Álvarez-Sabín et al. have reported on the phenomenon of delayed traumatic ICH^17^, studies that demonstrate a variance in the frequency of ICH progression according to the type of ICH are lacking. Additionally, systematic clinical analyses on the influence of each ICH type on patient prognosis remain unexplored. The ICH type characterized in this study as petechial hemorrhage has also been referred to as “blossomed” or exhibiting a “salt and pepper appearance” in prior research^16,18^. Pathologically, this phenotype signifies a severe manifestation of traumatic subarachnoid hemorrhage that extends into the brain parenchyma, arising from progressive microvascular rupture and consequent bleeding. We hypothesized that PH type would have the worst prognosis due to these pathological differences, and this was confirmed by the XGboost model's feature importance analysis.

### Skull fracture and counter coup injury

The clinical significance of counter coup head injury, characterized by brain injury occurring on the side opposite to the point of impact, has been suggested as a potential indicator of the severity of head trauma^19^. This perspective is based on the understanding that counter coup injuries are frequently associated with a higher risk of complications, including brain swelling and bleeding, compared to injuries that occur solely at the site of impact, known as coup injuries^9^.

In this study, we observed that the incidence of counter coup ICH was 17.9% in patients with occipital fractures, a rate higher than in patients with skull fractures at other locations (3.7% in frontal fractures, 7.2% in temporal fractures, and 3.7% in parietal fractures). This led to a notably increased frequency of ICH in the frontal lobe among patients whose initial impact was on the occipital skull. This observed trend may be linked to brain contusions that occur on the irregular surfaces of the anterior cranial fossa of the skull and structures like the anterior clinoid process. This could account for the prevalent association of counter coup ICH in the frontal lobe with TBIs involving occipital skull impacts^9^.

In our study, we successfully developed an algorithm capable of predicting an individual's prognosis using CT findings and clinical information. By integrating both clinical and radiological factors, such as counter coup injury and the specific type of ICH, we achieved high accuracy in predicting ICH progression among patients with mild to moderate traumatic brain injury (TBI).

The proposed XGBoost model demonstrated an average accuracy of 91% in predicting ICH progression, surpassing the logistic regression model, which achieved an AUC of 0.82. This enhanced performance emphasizes the efficacy of the XGBoost model in predicting ICH progression, highlighting the benefits of applying advanced machine learning techniques over traditional statistical methods for clinical predictions. Furthermore, our analysis validated the significant utility of SHAP values derived from the XGBoost model in assessing individual ICH progression risks. The incorporation of SHAP values enhances the visualization of individual risk factors, offering clinicians a crucial tool for interpreting the effects of various predictors on ICH progression at a personalized level. This capability facilitates more precise and tailored clinical decision-making.

To the best of our knowledge, this study represents the first attempt to develop a machine-learning model specifically for predicting ICH progression using image data from CT scans. We anticipate that our findings will contribute to the early identification of patients at risk for ICH progression, thereby informing treatment decisions and monitoring strategies. This approach has the potential to mitigate the risk of complications and enhance overall outcomes in patients with traumatic brain injury (TBI).

### Study limitations

The current study is subject to several limitations. Firstly, due to the limited number of patients in each age group, we were unable to analyze the risk of ICH progression across different age demographics. Secondly, we did not account for the potential impact of variables such as current medication use and underlying health conditions on ICH progression in TBI patients. Due to the challenges in obtaining a complete medical history from patients presenting to the emergency room with traumatic brain injury, our study focused primarily on factors that can be quickly and readily obtained in the ER, particularly radiological factors, to investigate their association with ICH progression. Although we investigated the history of antiplatelet and anticoagulation medication use, only a small proportion of patients (27 out of 650, or 4.2%) were confirmed to have used these medications. This limited number of patients was insufficient to establish a statistical correlation with ICH progression. This likely reflects the unreliability of initial medical history investigations and suggests that patients who were on antiplatelet or anticoagulation therapy might have presented with more severe ICH, thus potentially excluding them from this study due to their immediate need for surgical intervention.

Thirdly, our machine learning model was developed using data from a single institution, highlighting the need for future studies to perform general validation of the models with external datasets.

In forthcoming research, we aim to enhance the accuracy of our algorithm in predicting the progression of TBI. To improve the predictability of our current machine learning algorithm, it will be crucial to gather more comprehensive individual information from patient medical records. Furthermore, future research should investigate the factors influencing the necessity of surgery among patients exhibiting ICH progression, particularly focusing on changes in the Glasgow Coma Scale (GCS) following follow-up and the subsequent need for surgical intervention. Such analysis is anticipated to hold substantial clinical significance.

## Conclusion

In conclusion, our study demonstrates the effectiveness of the XGBoost model in predicting ICH progression in patients with traumatic brain injury (TBI). The application of this machine learning algorithm using the XGBoost model is anticipated to enhance the evaluation of prognosis and in-hospital management for TBI patients. Notably, we identified that patients presenting with a counter coup head injury and PH type ICH are at a significantly higher risk of ICH progression. Evaluating these two factors, derived from initial radiological assessments using head CT scans, showed substantial predictive accuracy for ICH progression.

The integration of features with substantial contributory influence, quantified using Shap values, has been shown to improve the overall predictive accuracy of our model. This study highlights the implementation of machine learning-based tools in the future, enhancing the care and management of patients with TBI.

### Supplementary Information

Supplementary Information.

## Data Availability

The authors confirm that the raw data supporting the findings of this study are available within its supplementary materials.
